# Phaeohyphomycosis caused by *Diaporthe phaseolorum* in an immunocompetent patient in Thailand: a case report

**DOI:** 10.1099/acmi.0.000128

**Published:** 2020-04-21

**Authors:** Kulwadee Laosakul, Sirida Youngchim, Mati Chuamanochan, Rujira Rujiwetpongstorn, Napatra Tovanabutra, Siri Chiewchanvit

**Affiliations:** ^1^​ Department of Internal Medicine, Faculty of Medicine, Chiang Mai University, Thailand; ^2^​ Division of Mycology, Department of Microbiology, Faculty of Medicine, Chiang Mai University, Thailand; ^3^​ Division of Dermatology, Department of Internal Medicine, Faculty of Medicine, Chiang Mai University, Thailand

**Keywords:** Black fungi, skin, *Diaporthe phaseolorum*, phaeohyphomycosis

## Abstract

Phaeohyphomycosis is caused by a large, heterogeneous group of darkly pigmented fungi. It is an infrequent infection in humans. However, the prevalence has been increasing in recent years especially in immunocompromised patients. *Diaporthe phaseolorum* is a common black fungal pathogen of plants, which rarely causes human infection. We report the first case of cutaneous infection caused by *Diaporthe phaseolorum* in an immunocompetent host and the first in Asia. Although, the review of the literature revealed two previous cases of cutaneous infection caused by this organism, both of them were in immunocompromised hosts. A slow-growing asymptomatic nodule was the major clinical feature. Histopathological examination showed granulomatous inflammation and pigmented septate hyphae and yeast-like cells. The fungal isolation was identified by morphological characteristics and DNA sequencing. The lesion was resolved after complete surgical excision and oral fluconazole for two months. This report highlights the potential role of *Diaporthe phaseolorum* as an emerging cause of infection in immunocompetent patients.

## Introduction

Cutaneous fungal infections are divided into three major groups: superficial, subcutaneous, and systemic [1]. Phaeohyphomycosis is an uncommon skin and subcutaneous tissue infection caused by dematiaceous fungi. However, in more recent years, its prevalence has increased especially in immunocompromised hosts. Rarely, cutaneous phaeohyphomycosis can occur in an immunocompetent host such as in our patient [2]. Clinical presentation can be a wide spectrum, ranging from papules, plaques, nodules to ulcers [2]. Infection can be limited to cutaneous or disseminated. When an infection is mucocutaneous, fungus inoculation usually results from direct trauma [2]. Lesions are usually asymptomatic, which may lead to the delay of detection. Extracutaneous involvement can rarely occur unless a patient is immunocompromised [2]. We report the first case of skin infection caused by *Diaporthe phaseolorum* in an immunocompetent host. To the best of our knowledge, this is the first record of cutaneous phaeohyphomycosis caused by this organism in Asia. This case highlights the importance of considering phaeohyphomycosis among the possible differential diagnoses in patients with skin nodules and the potential role of *Diaporthe phaseolorum* as an emerging cause of infection in immunocompetent patients.

## Case Report

A 66-year-old woman from Thailand presented with an asymptomatic slow-growing nodule on the dorsum of the right hand for three months. She denied a history of previous trauma. No ulceration or discharge was observed. There were no systemic symptoms. She was a hairdresser and lived in a rural area. She denied a history of contact with any plants. The patient had no special medical conditions except for allergic rhinitis. She took antihistamines occasionally. Neither immunosuppressive agents nor other medications were taken.

**Fig. 1.**
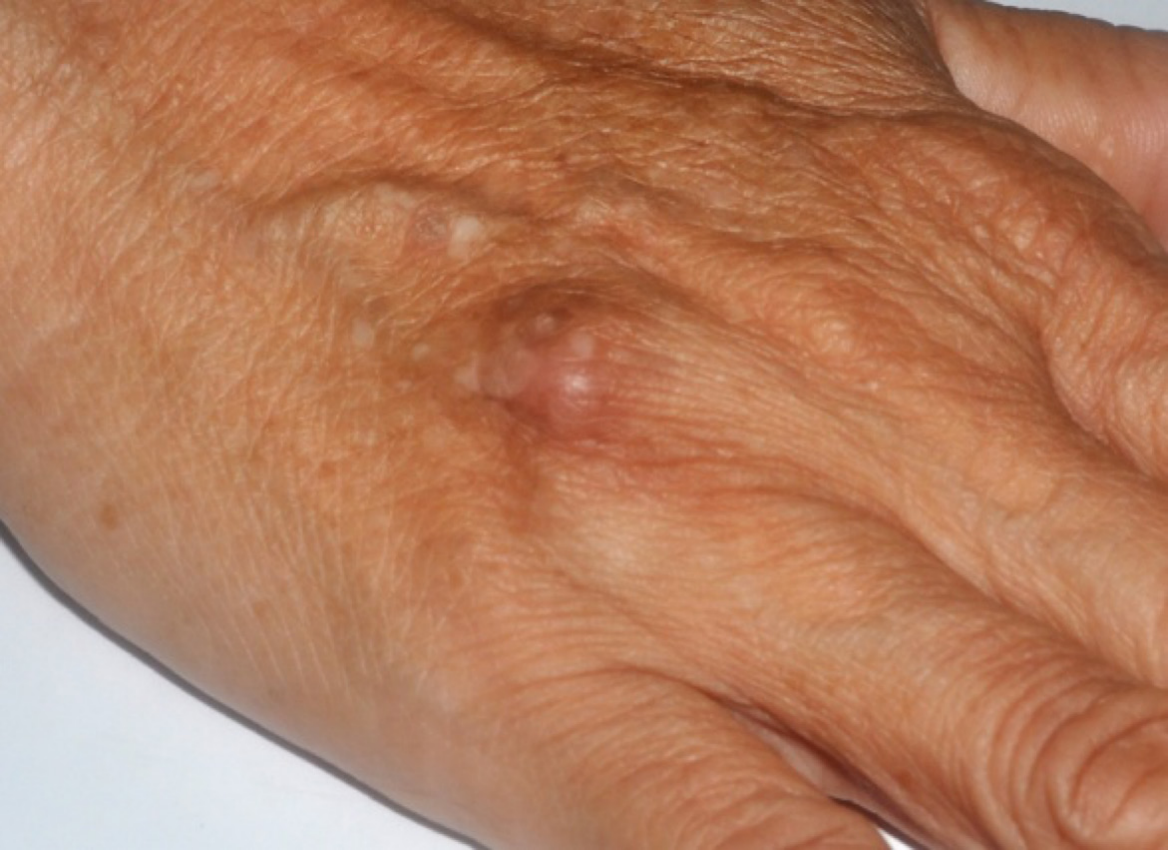
Well-circumscribed, hard, skin-colored dermal nodule, 2 cm in diameter on the dorsal aspect of the right hand of a patient

Physical examination revealed a solitary well-circumscribed, hard, mildly tender skin-colored dermal nodule, sized 2 cm in diameter on the dorsal aspect of the right hand (Fig. 1). The nodule was not adherent to overlying skin. No regional lymphadenopathy was found.

**Fig. 2.**
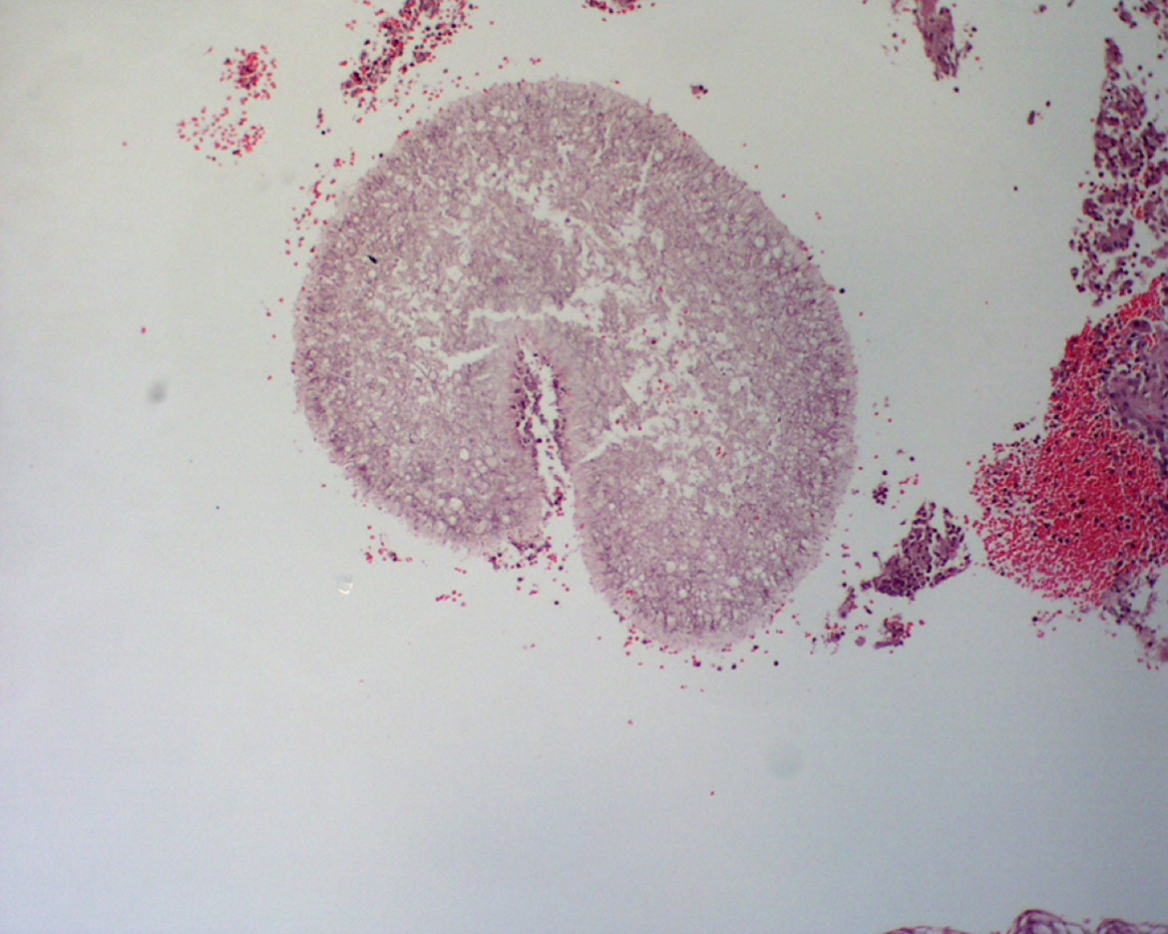
Fungal colonies with slightly pigmented septate hyphae and yeast-like cells (H and E, ×100).

**Fig. 3.**
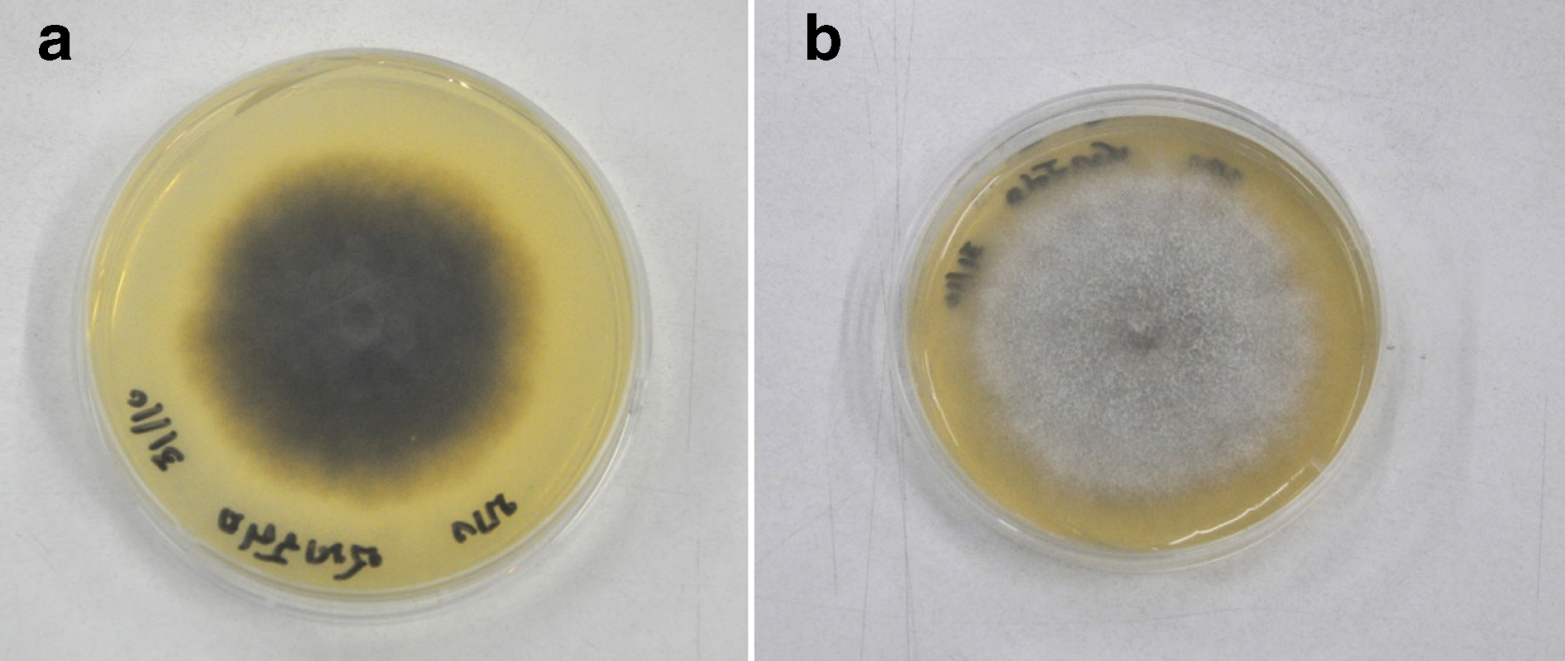
Mycological culture showed velvety greyish colony with black center.

The histopathologic examination of the skin lesion showed well-defined suppurative granulomatous inflammation in the deep dermal layer with normal epidermis and upper dermis and without sinus tract. Fungal colonies with slightly pigmented septate hyphae and yeast-like cells were seen (Fig. 2). Mycological culture from a biopsied specimen on Sabouraud's dextrose agar showed a velvety greyish colony with a black centered colony at 28 °C (Fig. 3a, b). Molecular identification of the causative agent was ascertained by PCR amplifying the internal transcribed spacer (ITS) 1 and 4 regions (ITS1 and ITS4) followed by bidirectional sequencing of the PCR product. A blast search of the ITS sequence data in Genebank at the NCBI website (http://www.ncbi.nlm.nih.gov/BLAST/) revealed it was 100 % identical to *Diaporthe phaseolorum* (accession number: MN788661) [3] (Supplementary material, available in the online version of this article). These confirmed the diagnosis of subcutaneous phaeohyphomycosis, caused by *Diaporthe phaseolorum*

The nodule was completely excised and the patient was treated with oral fluconazole 200 mg daily for two months. At the one-year follow-up visit, there was no recurrence of the skin lesion.

## Discussion

Phaeohyphomycosis is a rare skin and subcutaneous tissue infection caused by dematiaceous fungi (black fungi), which contain melanin in the cell walls. These fungi are usually found in a humid and dusty areas involving soil and plants. Therefore, this infection is usually seen in rural workers and gardeners, especially those with a history of previous trauma [2, 4]. Melanin is the main virulence factor in explaining the pathogenic potential of dematiaceous fungi. It destroys free radicals generated by the oxidative process within the hosts’ phagocytic cells and binds to hydrolytic enzymes to block phagocytic cell activity. Furthermore, melanin can inhibit the action of antifungal drugs [4–7]. The dominating etiological agents are *Alternaria* spp., *Bipolaris* spp., *Cladophialophora* spp., *Curvularia* spp., and *Phialophora* spp [2].

A recent international study presented that the most common predisposing factors for dematiaceous fungi infection were corticosteroid use and solid organ transplant. Local superficial infection usually developed in patients who have diabetes mellitus. While malignancy, chemotherapy, and neutropenia were frequent risk factors associated with local deep infection and disseminated infection [4], other predisposing factors include peritoneal dialysis, renal insufficiency, chronic granulomatous disease, Cushing syndrome, and HIV infection [2, 6]. Rarely, cutaneous phaeohyphomycosis can occur in an immunocompetent host such as in our patient.

Infection can be either mucocutaneous or disseminated. The wide variety of mucocutaneous lesions range from papules, plaques, pustules, nodules, cysts, abscesses, sporotrichoid lesions, and kaposiform lesions [2, 4, 8, 9]. They are usually asymptomatic and could be solitary or multiple [2]. The most frequent mechanism is traumatic inoculation, so the lesions are usually distributed on the exposed area e.g. extremities and face [1, 2]. Rarely, extracutaneous involvement can occur through local or blood dissemination from a focal cutaneous lesion or vascular entry [2]. Immunocompromised patients are mainly at risk of dissemination. Despite adequate treatment, the mortality rate of disseminated infection is 40–80 % [9].

According to the wide spectrum of cutaneous lesions, histopathologic and microscopic studies have a role in the diagnosis of cutaneous phaeohyphomycosis. In our case, multiple pigmented septate hyphae were clearly seen in the H and E section and were emphasized with Periodic acid-Schiff and Grocott-Gomori methenamine silver stains. The histopathology findings in this patient showed fungal colonies that were similar to the findings in mycetoma yet with normal epidermis and upper dermis without the sinus tract.

*Diaporthe phaseolorum* is a common plant fungal pathogen that rarely infects humans. The present case was the third case of human cutaneous infection caused by *Diaporthe phaseolorum* and the first in Asia. The first case was an HLTV1-infected farmer with eumycetoma with osteomyelitis of the forefoot [10], and the second case was a renal transplant Brazillian farmer with diabetes mellitus, who presented with a local granulomatous lesion [11]. As the previous two cases were immunosuppressed hosts, our present case was the first case of *Diaporthe phaseolorum* infection in an immunocompetent host. Another two cases of human invasive infection caused by *Diaporthe* spp. had been reported including an urban gardener with osteomyelitis of the finger [12] and a gardener with keratitis after rose thorn injury [13]. The epidemiology of these cases shows that this fungus preferentially infected people who have contact with plants, so this highlights the role of the plant in *Diaporthe* infection.

Although standard treatment of phaeohyphomycosis has not been established, complete surgical resection with systemic antifungal medications shows good efficacy. Itraconazole (400 mg day^–1^) is a widely used antifungal [2]. Voriconazole, posaconazole, and caspofungin also provide good results. The treatment duration depends on each case. It is recommended to continue the antifungal drug for a month after the lesions are healed or for 4 months after the lesions are excised. The duration for the lesion to heal ranges from 2 to 18 months [2].

The previous cases with *Diaporthe* cutaneous infection were successfully treated with oral itraconazole [10, 11]. The case of granulomatous lesion also underwent complete excision after receiving oral itraconazole for one month [11]. In our patient, the lesion was totally excised with additional antifungal therapy for two months. There was no recurrence of the lesion at a one-year follow-up visit.

In conclusion, we presented the first case of subcutaneous phaeohyphomycosis caused by *Diaporthe phaseolorum* in an immunocompetent host. This highlights the importance of considering phaeohyphomycosis among the possible differential diagnoses, even in an immunocompetent host, as they can be misdiagnosed for other soft tissue tumors or inflammation e.g. fibroma, lipoma, or foreign body reaction. Histopathologic examination, tissue cultures and molecular diagnosis are essential in the diagnosis of this condition. Complete surgical excision seems to be the best therapeutic option for solitary subcutaneous phaeohyphomycosis. To the best of our knowledge, this is the first record of cutaneous phaeohyphomycosis caused by this organism in Asia. This report features the potential role of *Diaporthe phaseolorum* as an emerging cause of infection in immunocompetent patients.

## Supplementary Data

Supplementary material 1

## References

[1] Bolognia JL, Schaffer JV, Cerroni L (2017). Dermatology.

[2] Caviedes MP, Torre AC, Eliceche ML, Valdivia Monteros DC, Volonteri VI (2017). Cutaneous phaeohyphomycosis. Int J Dermatol.

[3] Benson DA, Cavanaugh M, Clark K, Karsch-Mizrachi I, Lipman DJ (2013). Genbank. Nucleic Acids Res.

[4] Revankar SG, Baddley JW, Chen SC-A, Kauffman CA, Slavin M (2017). A mycoses study group international prospective study of phaeohyphomycosis: an analysis of 99 Proven/Probable cases. Open Forum Infect Dis.

[5] Revankar SG, Sutton DA (2010). Melanized fungi in human disease. Clin Microbiol Rev.

[6] Silveira F, Nucci M (2001). Emergence of black moulds in fungal disease: epidemiology and therapy. Curr Opin Infect Dis.

[7] Jacobson ES (2000). Pathogenic roles for fungal melanins. Clin Microbiol Rev.

[8] Tirico MCCP, Neto CF, Cruz LL, Mendes-Sousa AF, Valkinir DEJ (2016). Clinical spectrum of phaeohyphomycosis in solid organ transplant recipients. JAAD Case Rep.

[9] Thomas E, Bertolotti A, Barreau A, Klisnick J, Tournebize P (2018). From phaeohyphomycosis to disseminated chromoblastomycosis: a retrospective study of infections caused by dematiaceous fungi. Med Mal Infect.

[10] Iriart X, Binois R, Fior A, Blanchet D, Berry A (2011). Eumycetoma caused by *Diaporthe phaseolorum* (*Phomopsis phaseoli*): a case report and a mini-review of *Diaporthe/Phomopsis* spp invasive infections in humans. Clin Microbiol Infect.

[11] Mattei AS, Severo CB, Guazzelli LS, Oliveira FM, Gené J (2013). Cutaneous infection by *Diaporthe phaseolorum* in Brazil. Med Mycol Case Rep.

[12] Sutton DA, Timm WD, Morgan-Jones G, Rinaldi MG (1999). Human phaeohyphomycotic osteomyelitis caused by the coelomycete *Phomopsis saccardo* 1905: criteria for identification, case history, and therapy. J Clin Microbiol.

[13] Mandell KJ, Colby KA (2009). Penetrating keratoplasty for invasive fungal keratitis resulting from a thorn injury involving Phomopsis species. Cornea.

