# Supplementary material for: Phaeohyphomycosis caused by Diaporthe phaseolorum in an immunocompetent patient in Thailand: a case report
Source: Access Microbiol. 2020 Apr 21;2(7):acmi000128. doi: 10.1099/acmi.0.000128 (PMC7497834; doi:10.1099/acmi.0.000128)
Supplement: Supplementary material 1 [file acmi-2-128-s001.pdf]

Culture and Molecular identification

ชนารัศมิ์ ศรีธาวิรัตน์ HN 2653789 SMI1637303

Specimen: Tissue biopsy

Fungal species information

Genus species : *Diaporthe phaseolorum*

MMC Code: MMC61D111

DNA Sequencing result

Primer	DNA products	DNA Sequencing
ITS1, ITS4	555 bp.	*Diaporthe phaseolorum*

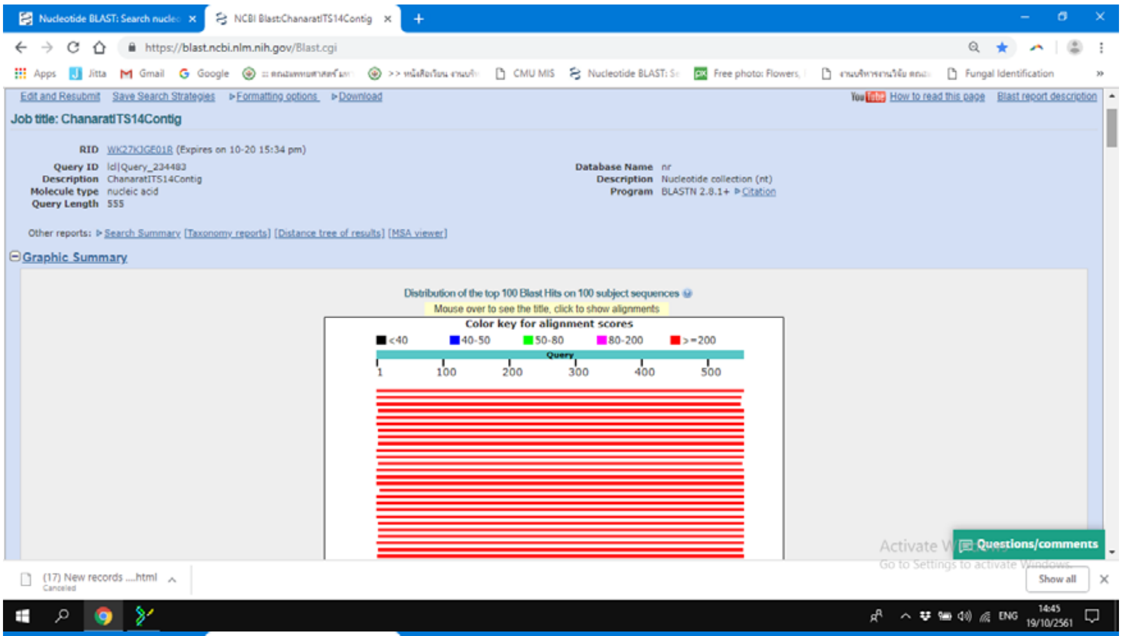

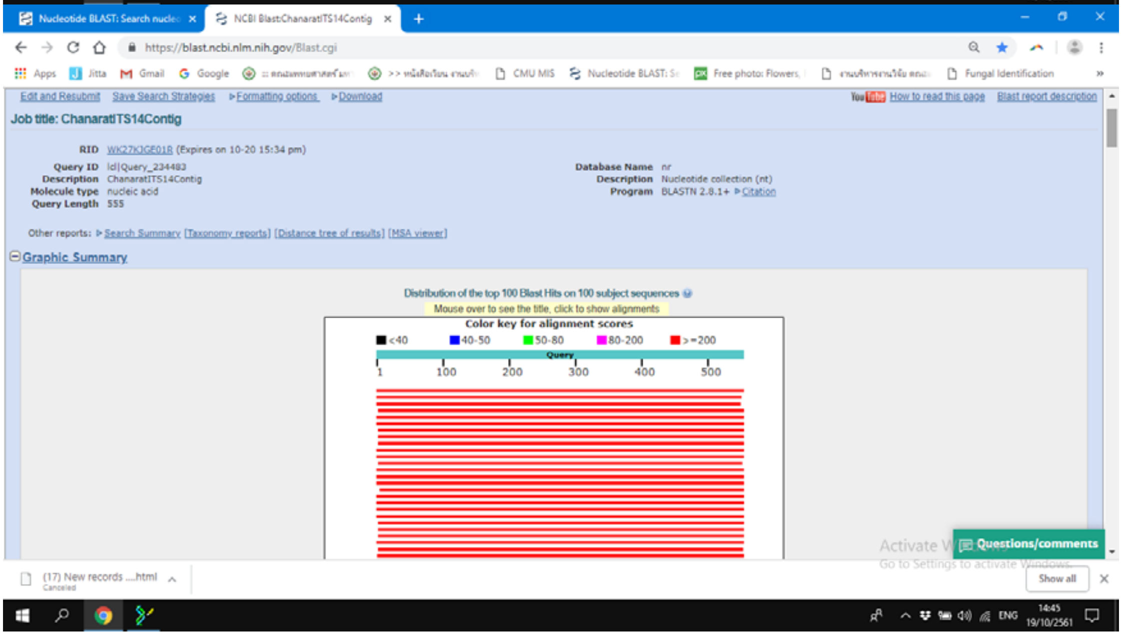

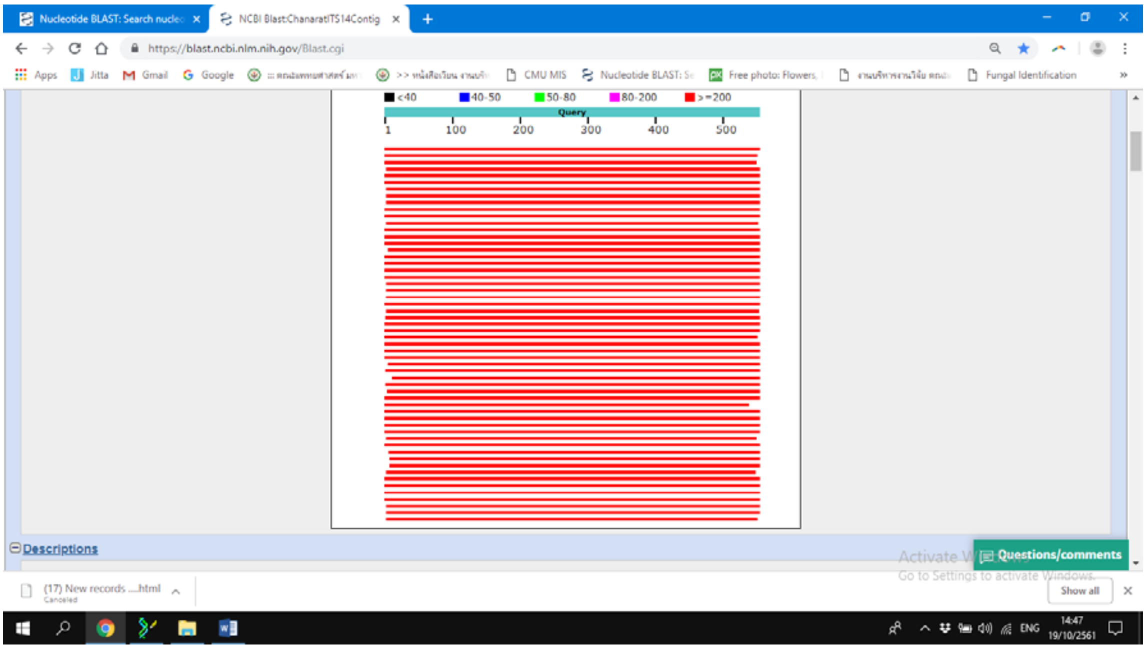

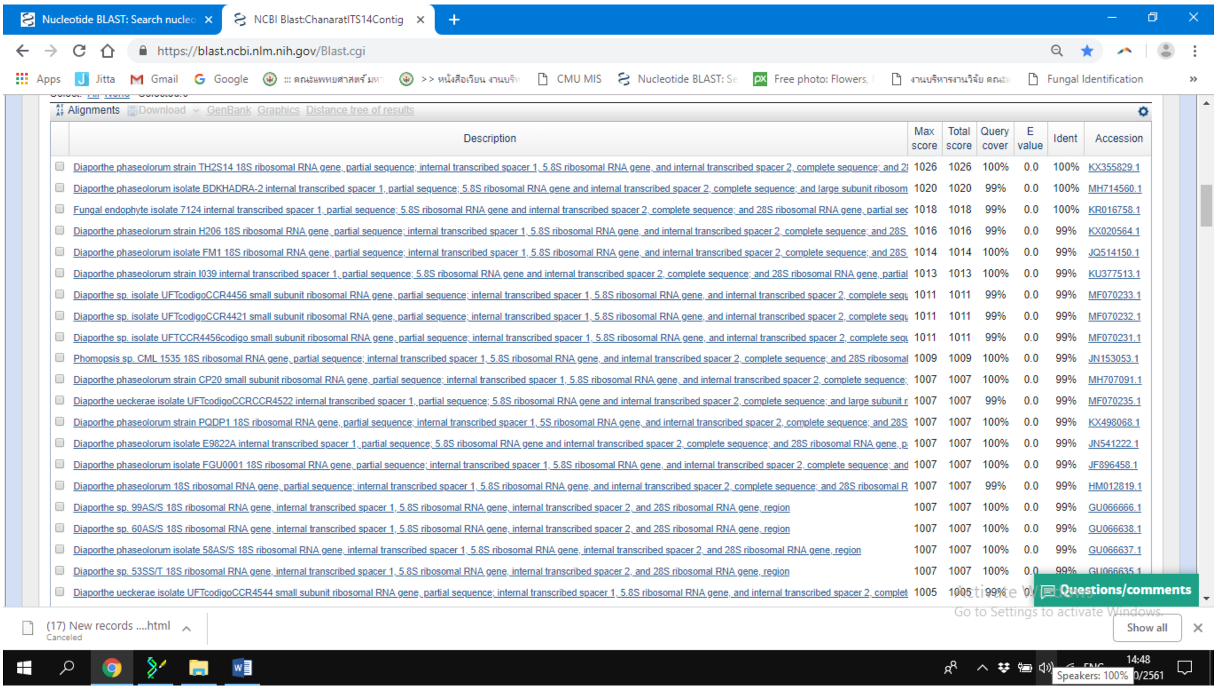

Description	Max score	Total score	Query cover	E value	Ident	Accession
Diaporthe phaseolorum strain TH2S14 18S ribosomal RNA gene, partial sequence; internal transcribed spacer 1, 5.8S ribosomal RNA gene, and internal transcribed spacer 2, complete sequence; and 2	1026	1026	100%	0.0	100%	KX355829.1
Diaporthe phaseolorum isolate BDKHADRA-2 internal transcribed spacer 1, partial sequence; 5.8S ribosomal RNA gene and internal transcribed spacer 2, complete sequence; and large subunit ribosom	1020	1020	99%	0.0	100%	MH714560.1
Fungal endophyte isolate 7124 internal transcribed spacer 1, partial sequence; 5.8S ribosomal RNA gene and internal transcribed spacer 2, complete sequence; and 28S ribosomal RNA gene, partial sec	1018	1018	99%	0.0	100%	KR016758.1
Diaporthe phaseolorum strain H206 18S ribosomal RNA gene, partial sequence; internal transcribed spacer 1, 5.8S ribosomal RNA gene, and internal transcribed spacer 2, complete sequence; and 28S	1016	1016	99%	0.0	99%	KX020564.1
Diaporthe phaseolorum isolate FM1 18S ribosomal RNA gene, partial sequence; internal transcribed spacer 1, 5.8S ribosomal RNA gene, and internal transcribed spacer 2, complete sequence; and 28S	1014	1014	100%	0.0	99%	JQ514150.1
Diaporthe phaseolorum strain I039 internal transcribed spacer 1, partial sequence; 5.8S ribosomal RNA gene and internal transcribed spacer 2, complete sequence; and 28S ribosomal RNA gene, partial	1013	1013	100%	0.0	99%	KU377513.1
Diaporthe sp. isolate UFTcodigoCCR4456 small subunit ribosomal RNA gene, partial sequence; internal transcribed spacer 1, 5.8S ribosomal RNA gene, and internal transcribed spacer 2, complete sequ	1011	1011	99%	0.0	99%	MF070233.1
Diaporthe sp. isolate UFTcodigoCCR4421 small subunit ribosomal RNA gene, partial sequence; internal transcribed spacer 1, 5.8S ribosomal RNA gene, and internal transcribed spacer 2, complete sequ	1011	1011	99%	0.0	99%	MF070232.1
Diaporthe sp. isolate UFTCCR4456codigo small subunit ribosomal RNA gene, partial sequence; internal transcribed spacer 1, 5.8S ribosomal RNA gene, and internal transcribed spacer 2, complete sequ	1011	1011	99%	0.0	99%	MF070231.1
Phomopsis sp. CML 1535 18S ribosomal RNA gene, partial sequence; internal transcribed spacer 1, 5.8S ribosomal RNA gene, and internal transcribed spacer 2, complete sequence; and 28S ribosomal	1009	1009	100%	0.0	99%	JN153053.1
Diaporthe phaseolorum strain CP20 small subunit ribosomal RNA gene, partial sequence; internal transcribed spacer 1, 5.8S ribosomal RNA gene, and internal transcribed spacer 2, complete sequence;	1007	1007	100%	0.0	99%	MH707091.1
Diaporthe ueckerae isolate UFTcodigoCCRCCR4522 internal transcribed spacer 1, partial sequence; 5.8S ribosomal RNA gene and internal transcribed spacer 2, complete sequence; and large subunit r	1007	1007	99%	0.0	99%	MF070235.1
Diaporthe phaseolorum strain PQDP1 18S ribosomal RNA gene, partial sequence; internal transcribed spacer 1, 5S ribosomal RNA gene, and internal transcribed spacer 2, complete sequence; and 28S	1007	1007	100%	0.0	99%	KX498068.1
Diaporthe phaseolorum isolate E9822A internal transcribed spacer 1, partial sequence; 5.8S ribosomal RNA gene and internal transcribed spacer 2, complete sequence; and 28S ribosomal RNA gene, p	1007	1007	100%	0.0	99%	JN541222.1
Diaporthe phaseolorum isolate FGU0001 18S ribosomal RNA gene, partial sequence; internal transcribed spacer 1, 5.8S ribosomal RNA gene, and internal transcribed spacer 2, complete sequence; and	1007	1007	100%	0.0	99%	JF896458.1
Diaporthe phaseolorum 18S ribosomal RNA gene, partial sequence; internal transcribed spacer 1, 5.8S ribosomal RNA gene, and internal transcribed spacer 2, complete sequence; and 28S ribosomal R	1007	1007	99%	0.0	99%	HM012819.1
Diaporthe sp. 99AS/S 18S ribosomal RNA gene, internal transcribed spacer 1, 5.8S ribosomal RNA gene, internal transcribed spacer 2, and 28S ribosomal RNA gene, region	1007	1007	100%	0.0	99%	GU066666.1
Diaporthe sp. 60AS/S 18S ribosomal RNA gene, internal transcribed spacer 1, 5.8S ribosomal RNA gene, internal transcribed spacer 2, and 28S ribosomal RNA gene, region	1007	1007	100%	0.0	99%	GU066638.1
Diaporthe phaseolorum isolate 58AS/S 18S ribosomal RNA gene, internal transcribed spacer 1, 5.8S ribosomal RNA gene, internal transcribed spacer 2, and 28S ribosomal RNA gene, region	1007	1007	100%	0.0	99%	GU066637.1
Diaporthe sp. 53SS/T 18S ribosomal RNA gene, internal transcribed spacer 1, 5.8S ribosomal RNA gene, internal transcribed spacer 2, and 28S ribosomal RNA gene, region	1007	1007	100%	0.0	99%	GU066635.1
Diaporthe ueckerae isolate UFTcodigoCCR4544 small subunit ribosomal RNA gene, partial sequence; internal transcribed spacer 1, 5.8S ribosomal RNA gene, and internal transcribed spacer 2, complet	1005	1005	99%	[illegible]	[illegible]	[illegible]

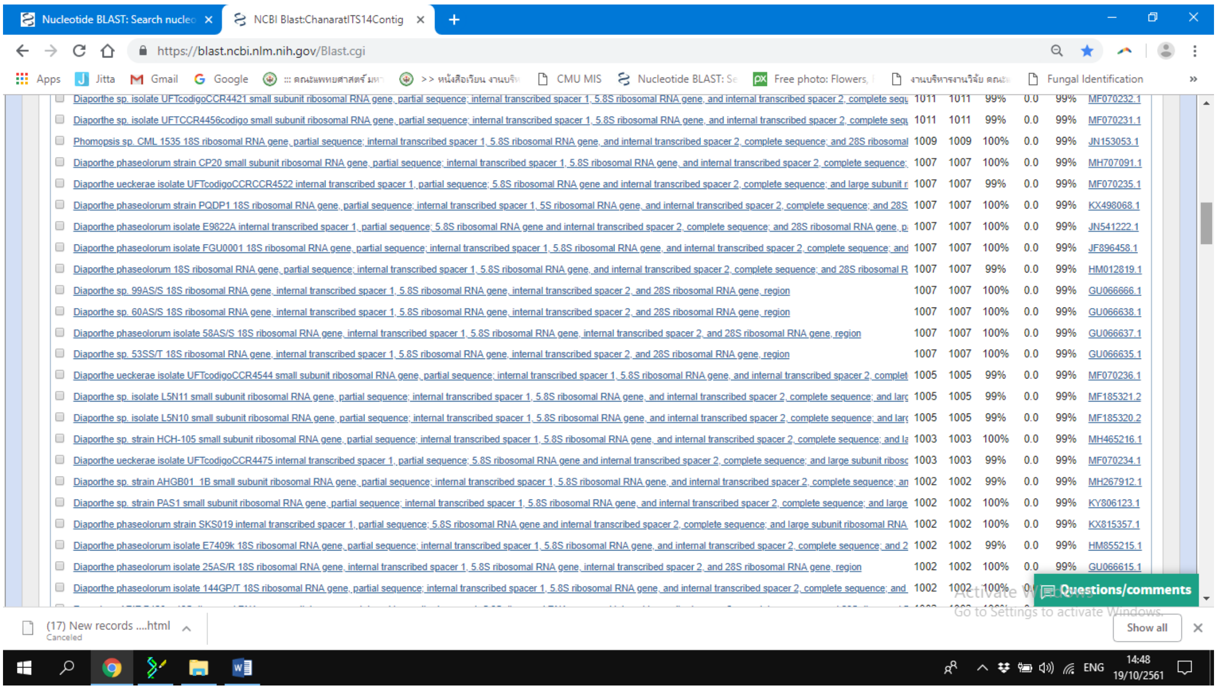

Diaporthe sp. isolate UFTcodigoCCR4421 small subunit ribosomal RNA gene, partial sequence; internal transcribed spacer 1, 5.8S ribosomal RNA gene, and internal transcribed spacer 2, complete sequ	1011	1011	99%	0.0	99%	MF070232.1
Diaporthe sp. isolate UFTCCR4456codigo small subunit ribosomal RNA gene, partial sequence; internal transcribed spacer 1, 5.8S ribosomal RNA gene, and internal transcribed spacer 2, complete sequ	1011	1011	99%	0.0	99%	MF070231.1
Phomopsis sp. CML 1535 18S ribosomal RNA gene, partial sequence; internal transcribed spacer 1, 5.8S ribosomal RNA gene, and internal transcribed spacer 2, complete sequence; and 28S ribosomal	1009	1009	100%	0.0	99%	JN153053.1
Diaporthe phaseolorum strain CP20 small subunit ribosomal RNA gene, partial sequence; internal transcribed spacer 1, 5.8S ribosomal RNA gene, and internal transcribed spacer 2, complete sequence;	1007	1007	100%	0.0	99%	MH707091.1
Diaporthe ueckerae isolate UFTcodigoCCRCCR4522 internal transcribed spacer 1, partial sequence; 5.8S ribosomal RNA gene and internal transcribed spacer 2, complete sequence; and large subunit r	1007	1007	99%	0.0	99%	MF070235.1
Diaporthe phaseolorum strain PQDP1 18S ribosomal RNA gene, partial sequence; internal transcribed spacer 1, 5S ribosomal RNA gene, and internal transcribed spacer 2, complete sequence; and 28S	1007	1007	100%	0.0	99%	KX498068.1
Diaporthe phaseolorum isolate E9822A internal transcribed spacer 1, partial sequence; 5.8S ribosomal RNA gene and internal transcribed spacer 2, complete sequence; and 28S ribosomal RNA gene, p	1007	1007	100%	0.0	99%	JN541222.1
Diaporthe phaseolorum isolate FGU0001 18S ribosomal RNA gene, partial sequence; internal transcribed spacer 1, 5.8S ribosomal RNA gene, and internal transcribed spacer 2, complete sequence; and	1007	1007	100%	0.0	99%	JF896458.1
Diaporthe phaseolorum 18S ribosomal RNA gene, partial sequence; internal transcribed spacer 1, 5.8S ribosomal RNA gene, and internal transcribed spacer 2, complete sequence; and 28S ribosomal R	1007	1007	99%	0.0	99%	HM012819.1
Diaporthe sp. 99AS/S 18S ribosomal RNA gene, internal transcribed spacer 1, 5.8S ribosomal RNA gene, internal transcribed spacer 2, and 28S ribosomal RNA gene, region	1007	1007	100%	0.0	99%	GU066666.1
Diaporthe sp. 60AS/S 18S ribosomal RNA gene, internal transcribed spacer 1, 5.8S ribosomal RNA gene, internal transcribed spacer 2, and 28S ribosomal RNA gene, region	1007	1007	100%	0.0	99%	GU066638.1
Diaporthe phaseolorum isolate 58AS/S 18S ribosomal RNA gene, internal transcribed spacer 1, 5.8S ribosomal RNA gene, internal transcribed spacer 2, and 28S ribosomal RNA gene, region	1007	1007	100%	0.0	99%	GU066637.1
Diaporthe sp. 53SS/T 18S ribosomal RNA gene, internal transcribed spacer 1, 5.8S ribosomal RNA gene, internal transcribed spacer 2, and 28S ribosomal RNA gene, region	1007	1007	100%	0.0	99%	GU066635.1
Diaporthe ueckerae isolate UFTcodigoCCR4544 small subunit ribosomal RNA gene, partial sequence; internal transcribed spacer 1, 5.8S ribosomal RNA gene, and internal transcribed spacer 2, complet	1005	1005	99%	0.0	99%	MF070236.1
Diaporthe sp. isolate L5N11 small subunit ribosomal RNA gene, partial sequence; internal transcribed spacer 1, 5.8S ribosomal RNA gene, and internal transcribed spacer 2, complete sequence; and larg	1005	1005	99%	0.0	99%	MF185321.2
Diaporthe sp. isolate L5N10 small subunit ribosomal RNA gene, partial sequence; internal transcribed spacer 1, 5.8S ribosomal RNA gene, and internal transcribed spacer 2, complete sequence; and larg	1005	1005	99%	0.0	99%	MF185320.2
Diaporthe sp. strain HCH-105 small subunit ribosomal RNA gene, partial sequence; internal transcribed spacer 1, 5.8S ribosomal RNA gene, and internal transcribed spacer 2, complete sequence; and la	1003	1003	100%	0.0	99%	MH465216.1
Diaporthe ueckerae isolate UFTcodigoCCR4475 internal transcribed spacer 1, partial sequence; 5.8S ribosomal RNA gene and internal transcribed spacer 2, complete sequence; and large subunit ribosc	1003	1003	99%	0.0	99%	MF070234.1
Diaporthe sp. strain AHGB01_1B small subunit ribosomal RNA gene, partial sequence; internal transcribed spacer 1, 5.8S ribosomal RNA gene, and internal transcribed spacer 2, complete sequence; an	1002	1002	99%	0.0	99%	MH267912.1
Diaporthe sp. strain PAS1 small subunit ribosomal RNA gene, partial sequence; internal transcribed spacer 1, 5.8S ribosomal RNA gene, and internal transcribed spacer 2, complete sequence; and large	1002	1002	100%	0.0	99%	KY806123.1
Diaporthe phaseolorum strain SKS019 internal transcribed spacer 1, partial sequence; 5.8S ribosomal RNA gene and internal transcribed spacer 2, complete sequence; and large subunit ribosomal RNA	1002	1002	100%	0.0	99%	KX815357.1
Diaporthe phaseolorum isolate E7409k 18S ribosomal RNA gene, partial sequence; internal transcribed spacer 1, 5.8S ribosomal RNA gene, and internal transcribed spacer 2, complete sequence; and 2	1002	1002	99%	0.0	99%	HM855215.1
Diaporthe phaseolorum isolate 25AS/R 18S ribosomal RNA gene, internal transcribed spacer 1, 5.8S ribosomal RNA gene, internal transcribed spacer 2, and 28S ribosomal RNA gene, region	1002	1002	100%	0.0	99%	GU066615.1
Diaporthe phaseolorum isolate 144GP/T 18S ribosomal RNA gene, partial sequence; internal transcribed spacer 1, 5.8S ribosomal RNA gene, and internal transcribed spacer 2, complete sequence; and	1002	1002	100%	[illegible]	[illegible]	[illegible]

cap_ITS1Chanarat 5-
TGATATGCTTAAGTTCAGCGGGTATTCCTACCTGATCC
GAGGTCAAATTTTCAGAAGTTGGGGGTTTAACGGCAGGGC
ACCGCCAGGGCCTTCCAGAACGAGATATAACTACTACGCT
CGGGGTCCTAGCGAGCTCGCCACTAGATTTCAGGGCCTG
CCCTCGTTAGAAGGCAGTGCCCCATCACCAAGCCAGGCTT
GAGGGTTGAAATGACGCTCGAACAGGCATGCCCTCCGGA
ATACCAGAGGGCGCAATGTGCGTTCAAAGATTCGATGATT
CACTGAATTCTGCAATTCACATTACTTATCGCATTTCGCTG
CGTTCTTCATCGATGCCAGAACCAAGAGATCCGTTGTTGAA
AGTTTTGATTCATTTATGTTTTTTACTCAGAGATTCACTATA
GAAACAAGAGTTTAGTTGGCCGCCGGCGGGCTGCTCCCT
GTTTCCAGGGGGCCTCAGTGAAGAGGCCGGCCTGCGCCG
AGGCAACAGATAGGTATAAGTTCACAAAGGG
cap_ITS4Chanarat 5-..TCAA-
TTTTCAGAAGTTGGGGGTTTAACGGCAGGGCACCGCCAGG
GCCTTCCAGAACGAGATATAACTACTACGCTCGGGGTCCT

AGCGAGCTCGCCACTAGATTTCAGGGCCTGCCCTCGTTAG
AAGGCAGTGCCCCATCACCAAGCCAGGCTTGAGGGTTGAA
ATGACGCTCGAACAGGCATGCCCTCCGGAATACCAGAGG
GCGCAATGTGCGTTCAAAGATTCGATGATTCACTGAATTCT
GCAATTCACATTACTTATCGCATTTCGCTGCGTTCTTCATC
GATGCCAGAACCAAGAGATCCGTTGTTGAAAGTTTTGATTC
ATTTATGTTTTTTACTCAGAGATTCACTATAGAAACAAGAGT
TTAGTTGGCCGCCGGCGGGCTGCTCCCTGTTTCCAGGGG
GCCTCAGTGAAGAGGCCGGCCTGCGCCGAGGCAACAGAT
AGGTATAAGTTCACAAAGGGTTTCTGGGTGCGCCGAAGCG
CGTTCCAGCAATGATCCCTCCGCAGG

Contig-0

TGATATGCTTAAGTTCAGCGGGTATTCCTACCTGATCC
GAGGTCAAATTTTCAGAAGTTGGGGGTTTAACGGCAGGGC
ACCGCCAGGGCCTTCCAGAACGAGATATAACTACTACGCT
CGGGGTCCTAGCGAGCTCGCCACTAGATTTCAGGGCCTG
CCCTCGTTAGAAGGCAGTGCCCCATCACCAAGCCAGGCTT
GAGGGTTGAAATGACGCTCGAACAGGCATGCCCTCCGGA
ATACCAGAGGGCGCAATGTGCGTTCAAAGATTCGATGATT
CACTGAATTCTGCAATTCACATTACTTATCGCATTTCGCTG
CGTTCTTCATCGATGCCAGAACCAAGAGATCCGTTGTTGAA
AGTTTTGATTCATTTATGTTTTTTACTCAGAGATTCACTATA
GAAACAAGAGTTTAGTTGGCCGCCGGCGGGCTGCTCCCT
GTTTCCAGGGGGCCTCAGTGAAGAGGCCGGCCTGCGCCG
AGGCAACAGATAGGTATAAGTTCACAAAGGGTTTCTGGGT
GCGCCGAAGCGCGTTCCAGCAATGATCCCTCCGCAGG
